# Back Pain and Body Posture Evaluation Instrument for Children and Adolescents (BackPEI-CA): Expansion, Content Validation, and Reliability

**DOI:** 10.3390/ijerph19031398

**Published:** 2022-01-27

**Authors:** Bruna Nichele da Rosa, Cláudia Tarragô Candotti, Luiza Rampi Pivotto, Matias Noll, Marcelle Guimarães Silva, Adriane Vieira, Jefferson Fagundes Loss

**Affiliations:** 1 Escola de Educação Física, Fisioterapia e Dança, Universidade Federal do Rio Grande do Sul, Porto Alegre 90690-200, Brazil; claudia.candotti@ufrgs.br (C.T.C.); luizarpivotto@gmail.com (L.R.P.); maguimaraess@hotmail.com (M.G.S.); adriane.vieira@gmail.com (A.V.); jefferson.loss@ufrgs.br (J.F.L.); 2 Postgraduate Program in Health Sciences, Faculdade de Medicina, Universidade Federal de Goiás, Goiânia 74605-050, Brazil; matias.noll@ifgoiano.edu.br; 3 Campus Ceres, Instituto Federal Goiano, Ceres 76300-000, Brazil; 4 Department of Sports Science and Clinical Biomechanics, University of Southern Denmark, 5230 Odense, Denmark

**Keywords:** back pain, neck pain, posture, surveys and questionnaires

## Abstract

The Back Pain and Body Posture Evaluation Instrument (BackPEI) was created in 2013 to assess back pain and its risk factors in school children. However, it does not assess neck pain or the habits of mobile device usage, which are aspects that are often part of school children’s lives. Therefore, we aimed to update the BackPEI questionnaire to include new questions assessing aspects related to neck pain and the use of mobile devices and to test the content validity and reliability of the new questions. The updated questionnaire was named Back Pain and Body Posture Evaluation Instrument for Children and Adolescents (BackPEI-CA). The content was validated by eight experts using the content validity index (CVI). To assess reliability, the BackPEI-CA questionnaire was applied at two different times in 105 school children, and Cohen’s kappa (k) and intraclass correlation coefficient (ICC) were calculated. All aspects assessed regarding content validity had a CVI higher than 0.8. The new questions presented moderate and good kappa values and excellent ICC values. The updated version of BackPEI-CA can be used as a clinic tool for assessing the presence, frequency, and intensity of back and neck pain and their risk factors.

## 1. Introduction

Research on back pain and its risk factors has increased in recent years due to the number of people suffering from issues related to the spine. The prevalence of back pain is high not only among adults but also among children and adolescents [1,2,3]. In 2013, the Back Pain and Body Posture Evaluation Instrument (BackPEI) was created [4]. Initially, the BackPEI was developed to assess the presence, frequency, and intensity of back pain and its risk factors (behavioral, postural, and demographic factors) among children and adolescents [4]. Because of its characteristics, the BackPEI has been widely used in research involving school children [5,6,7,8,9,10] and has been adapted for use in other countries [11,12].

The BackPEI, however, does not assess aspects related to neck pain [4], one of the most frequent musculoskeletal pains among adolescents, with an incidence of 28% [13]. Moreover, it is considered the eighth highest cause of disability among adolescents aged between 15 and 19 years, according to the World Health Organization [13]. In addition, the physical, social, and mental impacts of neck pain can persist throughout adulthood [13]. Thus, tools assessing the presence of neck pain and its impact on children and their routines are important.

Among back pain-associated risk factors assessed by BackPEI are postural and behavioral habits [4] and common habits in school children’s’ routine, such as physical activity, habits related to watching television and using the computer, and posture adopted to carry school supplies, to sleep, to write in the classroom, and to lift objects from the ground [4]. However, in recent years, the use of mobile devices, such as smartphones and tablets, has become common among teenagers [14]. Mobile devices allow access to different tools anywhere and at any time [15]. In 2018, it was estimated that 95% of adolescents from the United States have access to smartphones [14]. This reality has been extended to other countries [15], and in recent years, research has been conducted to investigate the addiction to mobile device usage among adolescents in different countries [16].

With the increase in mobile device use, the association between the use of mobile devices and musculoskeletal pain has been investigated [17]. The hypothesis is that the posture adopted to use the smartphone could cause discomfort to the neck and shoulders [18,19]. “Text neck” is the term used to describe the association between neck posture during smartphone use and neck pain [18]. However, the information available on the contribution of mobile device use to neck pain development is inconclusive [15,18,19]. Thus, this subject requires further investigation and new tools to assess the habits of mobile device usage among children and adolescents.

In view of these changes in the life habits of children and adolescents, such as the inclusion of mobile devices in their routines, the BackPEI should be updated to keep up with new trends. The aim of this study was, therefore, to update the BackPEI questionnaire to include new questions that assess aspects related to neck pain and the use of mobile devices and to test the content validity and reproducibility of these new questions.

## 2. Materials and Methods

### 2.1. Questionnaire Update

The new version of BackPEI was named Back Pain and Body Posture Evaluation Instrument for Children and Adolescents (BackPEI-CA) because a version of BackPEI for adults was developed in 2017, namely the Back Pain and Body Posture Evaluation Instrument for Adults (BackPEI-A) [20]. Therefore, this new version was tailored to children and adolescents.

The questionnaire update followed five steps [4]: (1) literature review of PubMed, Medline, and Scopus databases to identify the posture adopted by children and adolescents when using mobile devices and to identify how neck pain is assessed among children and adolescents for inclusion in BackPEI-CA; (2) development of the first version of the BackPEI-CA questionnaire; (3) assessment of new questions by experts for content validation and elaboration of the final version of BackPEI-CA; (4) assessment of the reliability of the new questions; and (5) translation to of the new BackPEI-CA questions to English. The new questions related to neck pain were based on both the literature review and on a BackPEI-A assessment, an auto-applied questionnaire that assesses back pain, neck pain, and possible associated factors in adults [20].

**Figure 1:**
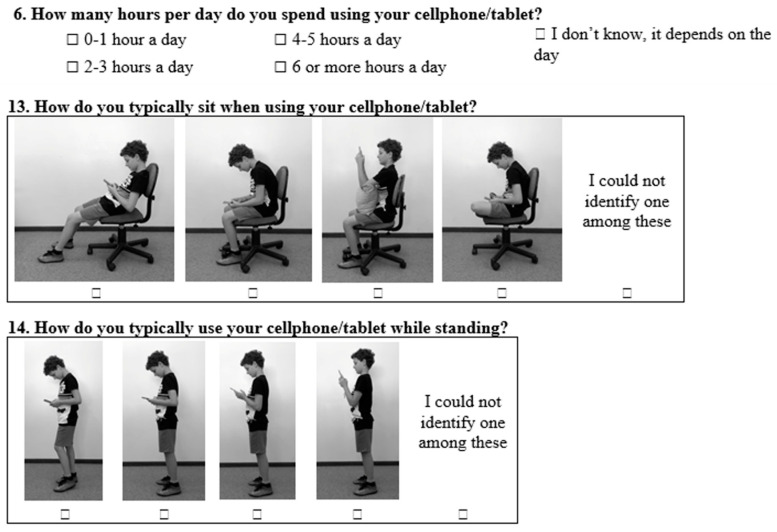
New questions (6, 13–14) assessing the use of mobile devices included in of the BackPEI-CA questionnaire.

**Figure 2:**
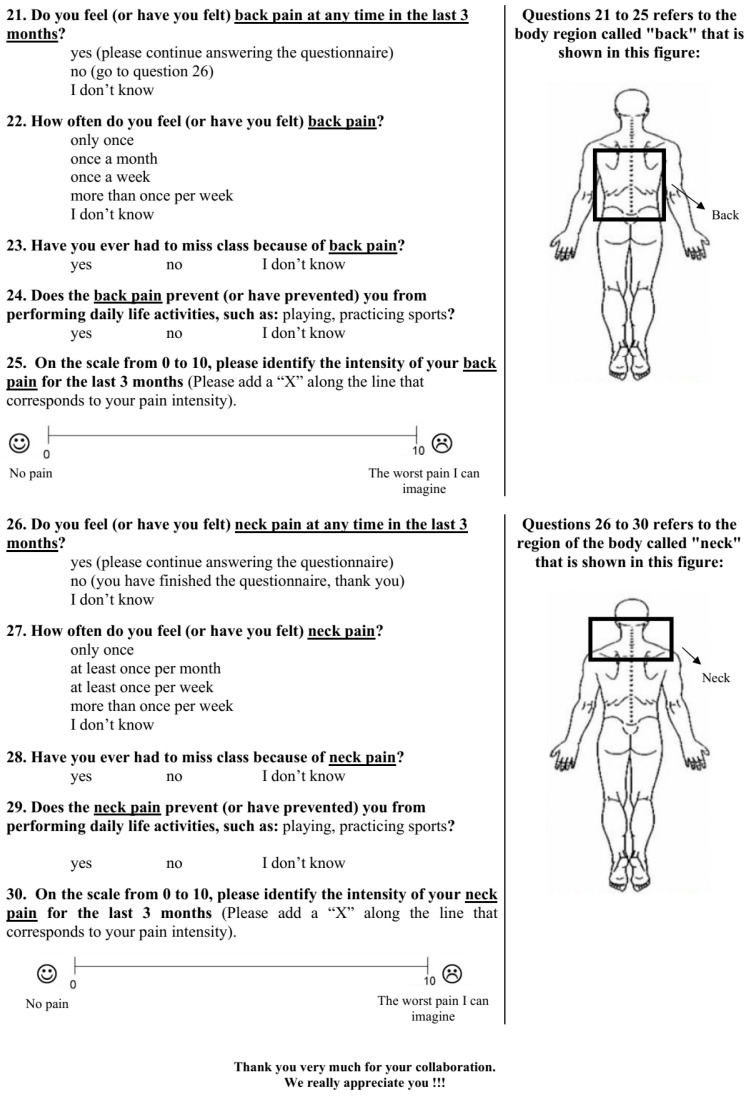
New graphic design to assess back pain (26–30) and the new questions (21–25) included in the BackPEI-CA questionnaire.

The BackPEI-CA contains 30 questions. Regarding the use of mobile devices, a new question about the daily time spent using mobile devices (question 6, Figure 1) was included, as were two new questions about the posture adopted during the use of mobile devices (questions 13 and 14, Figure 1). In relation to the assessment of back pain and neck pain (Figure 2), we included two new questions about the impact of back pain on children and adolescents’ lives (questions 23 and 24) and five new questions to assess neck pain (questions 26 to 30). The layout of questions about back pain (questions 21 to 25) was changed to include one new body region to assess pain and with a new graphic design to improve understanding of the body regions to which the questions were related.

Of the 30 questions, 28 were multiple-choice questions, for which the participants could choose only one option: the one that best represents their perceived condition. The remaining two questions (25 and 30) were related to back pain intensity and neck pain intensity and were answered using the visual analog pain scale (VAS). Questions 1 to 20 were related to the possible risk factors for back and neck pain. Questions 21 to 30 assessed the presence, frequency, and intensity of back and neck pain and if the pain prevented school attendance or play. In addition to the original version [4], BackPEI-CA has one female and one male version. Both updated versions (Supplementary Material) are available at www.ufrgs.br/biomec/materiais.htm (accessed on 29 November 2021). The updated version of BackPEI-CA was designed and applied only in the Portuguese language, the native language of study participants.

### 2.2. Content Validity

At this stage, eight experts were invited to participate. These experts were physical education teachers, physical therapists, chiropractors, and researchers in areas of body posture, public health, musculoskeletal pain, and biomechanics who had already used the original version of the BackPEI questionnaire.

The experts received the initial version of BackPEI-CA and a specific questionnaire for content validity to analyze the following aspects: (1) the clarity, ease of understanding, and general applicability of the new questions and (2) if the new questions allowed the identification of smartphones/tablet use behavior and adequately assessed back pain and neck pain. The experts could answer if the questionnaire was “Adequate,” “Not adequate,” or “Could improve” for each analyzed aspect. When the answer was “Not adequate” or “Could improve,” the questionnaire was revised following the experts’ suggestions and was returned for re-assessment. This procedure was repeated until an agreement was reached between the experts [4]. The content validity index (CVI) was used to identify agreement between experts. The CVI measures the agreement between experts related to a specific item of the tool. To calculate the CVI of each question, the total number of favorable responses (“Adequate” answers) was divided by the total number of answers, according to Equation (1). The acceptable agreement rate among experts to the validity of new questions should be at least 0.8 and preferably greater than 0.9 [21].

(1) $$\mathrm{CVI}=\frac{\;\mathrm{number}\;\mathrm{of}\;\mathrm{favorable}\;\mathrm{responses}}{\mathrm{total}\;\mathrm{number}\;\mathrm{of}\;\mathrm{responses}}$$

### 2.3. Reliability

To test the test-retest reliability, after content validity, the final version of the BackPEI-CA questionnaire was applied at two different times with an interval of 7 days. The questionnaire was initially distributed as a printed form in a private school located in the urban area of Porto Alegre, Rio Grande do Sul, Brazil. According to the census (2010), the municipality had approximately 1,400,000 inhabitants. The human development index (IDH) was 0.805 (the score for Brazil at the same time was 0.724) The school was intentionally selected, and the assessment occurred in November and December 2019 However, due to the COVID-19 pandemic, it was distributed as in an online form in March and April 2020. At the school, the data collection procedure was performed by a trained team formed by researchers. For the online assessment, the questionnaire was transformed into an easily accessible, online form with a friendly interface using Google Forms. The research was propagated using social networks, and the parents or guardians of prospective participants could contact the responsible researcher. The school children filled the questionnaire only after the parents’ or guardians’ approval. The parents or guardians signed the Consent Form both to the assessment at school and to the online assessment. Those that fulfilled the eligibility criteria were sent a link to the BackPEI-CA form. School children from the fifth grade of elementary school to the third year of high school (10–17 years) participated in this stage.

### 2.4. Sample

For test-retest reliability, the sample was randomized, and the sample size was determined according to Sim and Wright [22] for categorical variables and according to Walter, Eliasziw, and Donner [23] for continuous variables. To detect a kappa value of 0.8, we assumed a kappa null hypothesis value of 0.4 (that is, any value less than 0.4 must be considered unacceptable), power of 80%, and a worst-case scenario of 10% positive ratings. Thus, for categorical variables, a sample of at least 102 participants was necessary for acceptable reliability. To detect an ICC value of 0.6 for continuous variables, we assumed a significance level of 5%, a power of 80%, an ICC null hypothesis value of 0.4, and two assessments per individual, resulting in a sample of 87 participants.

School children aged between 10 and 17 years who were able to read and understand the questionnaire without assistance were eligible for this study, both on assessment at school and online assessment. Those who submitted incomplete answers and did not return the questionnaire on the second day of application were excluded. The study was approved by the Research Ethics Committee of the University (CAAE: 14563919.0.0000.5347).

### 2.5. Statistical Analysis

Experts’ answers related to content validity were tabulated, and the data were analyzed using Microsoft Excel software (version 2019, Microsoft Corporation, Washington, DC, USA). The answers of school children, related to both days of application (reliability stage), were tabulated and analyzed using the Statistical Package for the Social Sciences (version 26.0). The data of the test-retest procedure of new questions were analyzed using the percentage of agreement (%C), Cohen’s kappa, and weighted kappa (k) [22,24]. The %C was classified as poor (<0.3), weak (0.31–0.5), moderate (0.51–0.7), good (0.71–0.9), and excellent (0.91–1) [25]. The kappa values were classified according to Schlademann [26] as poor (<0.2), light (0.21–0.4), moderate (0.41–0.6), good (0.61–0.8), and very good (0.81–1). Questions with values of k ≥ 0.4 and %C > 50% were included in the updated version [27,28]. The agreement between the test and retest of questions about back pain intensity and neck pain intensity was measured using a two-way mixed effects, absolute agreement Intraclass Correlation Coefficient (ICC). The ICC values were classified as weak (<0.4), moderate (0.4–0.75), and excellent (>0.75) [24]. The significance level was set at *p* < 0.05.

## 3. Results

**Figure 3:**
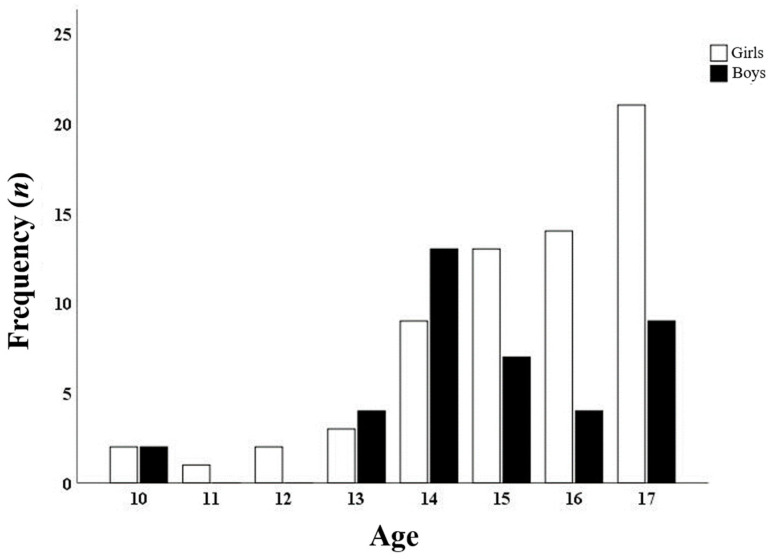
Distribution of school children by gender and age (Porto Alegre, Brazil, 2020).

BackPEI-CA was answered at least once by 153 school children. Of these, 48 did not answer on the second day of the assessment. Therefore, 105 school children were included in the test-retest reliability analysis. The mean age was 15.2 (±1.8) years, the mean body mass was 57.1 (±13.8) kg, the mean height was 162.2 (±11.2) cm, and 65 (61.9%) were girls. Figure 3 shows details of the sample by gender and age. Table 1 and Table 2 present the characteristics of school children assessed in relation to back pain, neck pain (Table 1), and habits of mobile device use (Table 2).

### 3.1. Content Validation

Two expert assessments were necessary to include new questions in the questionnaire. In the first assessment, some experts suggested necessary changes in the content of the new questions. Table 3 presents the aspects assessed in relation to the content validity of the new BackPEI-CA questions and the CVI results for each question. Of the seven aspects assessed regarding content validity, only two aspects obtained a CVI of less than 1. However, both aspects presented a CVI higher than 0.8 (Table 3).

### 3.2. Reliability

The test-retest reliability results are presented in Table 4 and Table 5. The new questions presented a k value ranging from 0.418 to 0.677 and were classified as moderate and good (Table 4); ICC values higher than 0.8 were considered excellent (Table 5).

## 4. Discussion

The self-administered questionnaire is an important assessment tool because of its ease of application, low cost, and possibility of self-reporting [4,26]. Therefore, it is one of the most used epidemiological study designs [26] for assessing aspects of public health, such as back pain. Hence, a variety of these questionnaires have been made available to assess aspects related to back pain [26,29,30].

To the best of our knowledge, apart from BackPEI-CA and BackPEI-A, no other questionnaire assesses aspects related to spine pain and the possible factors associated with the pain [4,29]. Furthermore, these questionnaires allow a range of back pain- and neck pain-related aspects to be assessed with only one tool. Despite its highly regarded profile [4], the original version of BackPEI is limited in terms of its inability to assess neck pain and habits related to mobile device usage, an important habit of children and adolescents. It is, therefore, important to keep the BackPEI questionnaire updated in relation to the main activities as well as the major pain complaints in school children.

Mobile device use is common, and it is estimated that in 2021, there were more than six billion smartphone users worldwide [30]. In a study that assessed 653 American adults, it was shown that participants spent approximately 1.5 h daily using a smartphone [31]. Among children and adolescents, this scenario is particularly common and is reflected in the sample of our study, in which 80% of assessed school children reported using smartphones or tablets for more than 2 h a day (Table 2). The amount of time spent on mobile devices varies among people of different ages due to the variety of uses, such as communication, internet, multimedia file access, and leisure [16]. Therefore, it is evident that there is an urgent need to add new questions assessing the habits of mobile device use.

Similarly, the rate of neck pain complaints has increased among school children. Neck pain is a major musculoskeletal complaint and the fourth cause of disability worldwide [32]. Among adolescents, it can reach a prevalence of up to 70% [33]. In the present study, the school children assessed (Table 1) presented a neck pain prevalence of 62.1%, and 18.5% reported disability as a difficulty in performing everyday tasks due to pain. These data reinforce the importance of updating the BackPEI questionnaire, which, with the addition of the new questions about neck pain, could be a useful tool for assessing two body regions (back and neck). We therefore proposed a new graphic design for BackPEI-CA (Figure 2) that illustrates which body region should be viewed as the “back” and which should be viewed as the “neck” to avoid confusion when answering the questions about pain.

The results showed that both the new graphic design and the new questions presented valid content (Table 3). The CVI ranged from 0.9 to 1, showing adequate agreement between the experts that assessed the content of the questions [21,34]. Thus, our BackPEI update is considered adequate to assess school children’s habits of using mobile devices and aspects related to back and neck pain. In addition, the new questions were also considered reliable because they presented moderate and good kappa values [26], adequate percentage of agreement [25], and excellent ICC values (Table 4). Therefore, BackPEI-CA, the updated version of the BackPEI questionnaire, can be a valid and reliable instrument to assess the presence, frequency, and intensity of back pain, neck pain, and the habits of using mobile devices, such as the posture adopted and the usage time. However, is important to emphasize that the questionnaire was originally designed and applied in the Portuguese language. Therefore, only the Portuguese version is considered a valid and reliable tool. Therefore, for it to be used to assess English-speaking users, we consider necessary a transcultural adaptation.

The limitations of this study are related to the inherent characteristics of closed questionnaires. That is, the options to answer new questions about the habits of using mobile devices did not present all possible options for posture adopted. However, we kept the alternative option of “I could not identify one among these” when the school children identified with another postural pattern so that they did not feel forced to fill in a pattern that was not their usual one. A second limitation of this study was the inclusion of the option “another way/I don’t know” on questions 13 and 14 because it does not allow the rater to know which is the most common pattern of school children. Moreover, our study presented a sample selection bias because the school children under 13 years old are in small number (<5 to each age); at ages 11 and 12, there were not any boys, and at ages over 15 years, there were a higher number of girls compared to the number of boys at the same age (Figure 3). However, despite these limitations, the BackPEI-CA can benefit epidemiological research and, in the clinical context, can act as a screening tool for school evaluation.

## 5. Conclusions

With its new graphic design and new questions that assess aspects of neck pain and mobile device usage habits, the BackPEI-CA questionnaire is a valid and reliable tool that can be used on clinical assessment or on school screening. Therefore, it allows for the of assessment of the presence, frequency, and intensity of back pain, neck pain, and associated risk factors, such as behavioral and postural habits.

## Figures and Tables

**Table 1:** Sample characteristics related to back pain and neck pain.

Back Pain	Frequency (%)	Neck Pain	Frequency (%)
Presence		Presence	
Yes	71 (67.6%)	Yes	64 (62.1%)
No	27 (25.7%)	No	36 (35%)
I don’t know	7 (6.7%)	I don’t know	3 (2.9%)
Total	105 (100%)	Total	103 (100%)
Frequency		Frequency	
Only once	24 (29.3%)	Only once	15 (22.7%)
At least once a month	23 (28%)	At least once a month	22 (33.3%)
At least once a week	24 (29.3%)	At least once a week	23 (34.8%)
I don’t know	11 (13.4%)	I don’t know	6 (9.2%)
Total	82 (100%)	Total	66 (100%)
Absenteeism at school		Absenteeism at school	
Yes	4 (43.9%)	Yes	5 (7.6%)
No	77 (93.9%)	No	59 (89.4%)
I don’t know	1 (1.2%)	I don’t know	2 (3%)
Total	82 (100%)	Total	66 (100%)
Disability		Disability	
Yes	16 (19.7%)	Yes	12 (18.5%)
No	64 (79.7%)	No	52 (80%)
I don’t know	1 (0.6%)	I don’t know	1 (1.5%)
Total	81 (100%)	Total	65 (100%)

**Table 2:** Sample characteristics related to habits of mobile device use.

**Daily Time of Mobile Devices Utilization**	
0 to 1 h	9 (8.6%)
2 to 3 h	33 (31.4%)
4 to 5 h	24 (22.9%)
6 h or more	27 (25.7%)
I don’t know	12 (11.4%)
Total	105 (100%)
**Posture Adopted to Use the Mobile Device in the Sitting Position**	
Adequate	34 (32.4%)
Inadequate	57 (54.3%)
I don’t know	14 (13.3%)
Total	105 (100%)
**Posture Adopted to Use the Mobile Device in the Standing Position**	
Adequate	56 (53.3%)
Inadequate	43 (41%)
I don’t know	6 (5.7%)
Total	100 (100%)

**Table 3:** Content validity aspects assessed by experts and the Content Validity Index (CVI) of each assessment.

BackPEI-CA Question	Item	CVI 1st Assessment	CVI 2nd Assessment
6, 13, and 14	Regarding the insertion of new questions, do you think it is possible to identify behavior during smart phones/tablet use?	0.6	0.9
13 and 14	Regarding the quality of images (photos) and adequation of postures used	0.9	1.0
23 and 24	Regarding the insertion of new questions about back pain, do you think they enable assessment of the impact of back pain on children’s and adolescents’ lives?	0.9	1.0
22	Regarding the adequacy of options to answer about the back pain frequency	0.6	1.0
21–25	Regarding the new graphic design of questions about back pain, do you think they facilitate the understanding that “back pain” refers to any thoracic and lumbar region?	0.8	1.0
26–30	Regarding the insertion of new questions about neck pain, do you think it is possible to identify the presence, frequency, and intensity of neck pain?	0.8	0.9
26–30	Regarding graphic design of questions about neck pain, do you think they facilitate the understanding that “neck pain” refers to any region of the cervical spine?	0.8	1.0

**Table 4:** Results of test-retest reliability of BackPEI-CA new questions.

Question	Question Description	*n*	Agreement (%)	Kappa (*p*)	95%CI	Bias
6	How many hours per day do you spend using your cellphone/tablet?	105	52.4	0.439 (<0.001)	0.309–0.569	−0.001
13	How do you typically sit when using your cellphone/tablet?	105	55.3	0.418 (<0.001)	0.288–0.551	−0.001
14	How do you typically use your cellphone/tablet while standing?	104	67.3	0.477 (<0.001)	0.342–0.596	−0.004
21	Have you felt (or been feeling) back pain in the last three months?	104	76	0.451 (<0.001)	0.286–0.598	−0.007
22	How often do you feel (or have you felt) back pain?	71	60.6	0.451 (<0.001)	0.330–0.554	−0.002
23	Have you ever missed any class because of the back pain?	71	81,7	0.559 (<0.001)	0.380–0.711	−0.008
24	Does the back pain prevent (or has it prevented) you from performing daily life activities, such as playing or practicing sports?	70	84.3	0.523 (<0.001)	0.265–0.746	−0.006
26	Have you felt (or been feeling) neck pain in the last three months?	103	77.5	0.556 (<0.001)	0.409–0.713	−0.001
27	How often do you feel (or have you felt) neck pain?	64	56.4	0.432 (<0.001)	0.311–0.561	−0.001
28	Have you ever missed any class because of the neck pain?	56	92.5	0.677 (<0.001)	0.364–0.913	−0.013
29	Does the neck pain prevent (or has it prevented) you from performing daily life activities, such as playing or practicing sports?	53	79.2	0.453 (<0.001)	0.214–0.679	−0.004

**Table 5:** Results of test-retest reliability of new continuous variables of BackPEI-CA.

Question	Question Description	*n*	ICC	95%CI	Test–Mean (SD)	Retest–Mean (SD)	SEM	MDC
25	Back pain intensity (cm)	70	0.828	0.828–0.933	5.2 (1.9)	5.1 (1.9)	0.81	1.58
30	Neck pain intensity (cm)	56	0.824	0.700–0.897	4.9 (2)	4.9 (2.3)	0.85	1.66

*n*, sample size; ICC, intraclass correlation coefficient; 95%CI, 95% confidence interval; SD, standard deviation; SEM, standard error of measurement; MDC, minimum detectable change.

## Data Availability

The data presented in this study are available on request from the corresponding author. The data is not publicly available as we do not have a public repository. But if you are interested, please contact Bruna (bruna.nichele@gmail.com) and we will send you the information.

## Supplementary Materials

The following supporting information can be downloaded at: https://www.mdpi.com/article/10.3390/ijerph19031398/s1, BackPEI-CA Questionnaire Female version; BackPEI-CA Questionnaire Male version.
