# Supplementary material for: Back Pain and Body Posture Evaluation Instrument for Children and Adolescents (BackPEI-CA): Expansion, Content Validation, and Reliability
Source: Int J Environ Res Public Health. 2022 Jan 27;19(3):1398. doi: 10.3390/ijerph19031398 (PMC8835607; doi:10.3390/ijerph19031398)
Supplement: Supplementary file 1 [file ijerph-19-01398-s001.zip › BackPEI-CA Male Version.pdf]

Back Pain and Body Posture Evaluation Instrument for Children and Adolescents (BackPEI-CA)

Dear Student!!!

Read each question carefully and then select the best answer from the choices provided.

If you have any question, please do not hesitate to ask the evaluator in charge.

Name:____ Date of birth:____/____/_____

Weight:____kg Height: _____cm School year: ☐6 ☐7 ☐8 ☐9 ☐10 (UK System)

Sex: ☐Male ☐Female School year: ☐5 ☐6 ☐7 ☐8 ☐9 (USA System)

Name of School:_______________________________________ City:_________________________

Father's name (or Guardian's):____

Mother's name (or Guardian's):____

---

1. Do you practice sport or physical exercise regularly at the school or outside the school?

☐ yes, please describe: _________________ ☐ no (go to question 4)

2. How many days per week do you practice sport or exercise?

☐ 1 – 2 days a week ☐ 5 or more days a week
☐ 3 – 4 days a week ☐ It varies by week

3. Do you practice this sport or physical exercise competitively?

☐ yes ☐ no

4. How many hours per day do you usually spend sitting watching television?

☐ 0 – 1 hour a day ☐ 6 – 7 hours a day
☐ 2 – 3 hours a day ☐ 8 hours or more a day
☐ 4 – 5 hours a day ☐ I don't know, it depends on the day

5. How many hours per day do you spend seated using your desktop/laptop computer?

☐ 0-1 hour a day ☐ 4-5 hours a day ☐ I don't know, it depends on the day
☐ 2-3 hours a day ☐ 6 or more hours a day

6. How many hours per day do you spend using your cellphone/tablet?

☐ 0-1 hour a day ☐ 4-5 hours a day ☐ I don't know, it depends on the day
☐ 2-3 hours a day ☐ 6 or more hours a day

7. Do you usually read or study in bed?

☐ yes ☐ no ☐ sometimes

8. What is your favorite sleeping position?

☐ on my side ☐ face up (on my back)
☐ face down (on my stomach) ☐ it varies

9. How many hours do you spend sleeping in a day - 24 hour period?

☐ 0 – 6 hours ☐ 8 – 9 hours ☐ I don't know, it depends on the day
☐ 7 hours ☐ 10 hours or more

10. How do you typically sit at your desk when writing while in school?

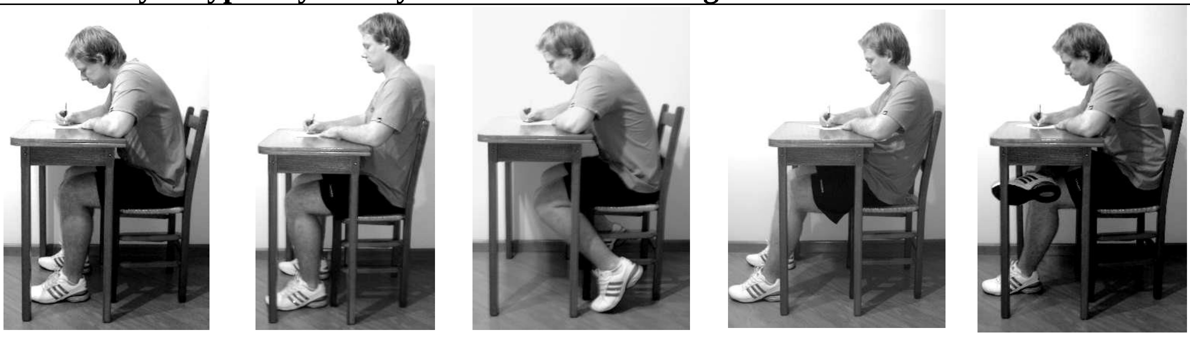

I could not identify one among these

☐ ☐ ☐ ☐ ☐ ☐

11. How do you typically sit on a chair or a bench when talking to your friends?

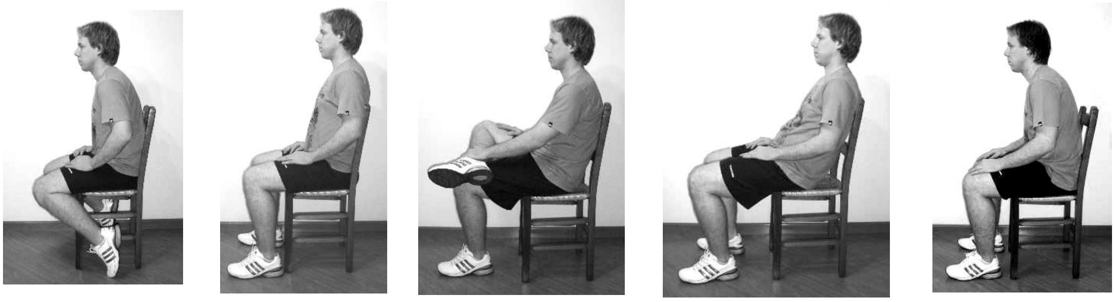

I could not identify one among these

☐ ☐ ☐ ☐ ☐ ☐

12. How do you typically sit when using your desktop or laptop computer?

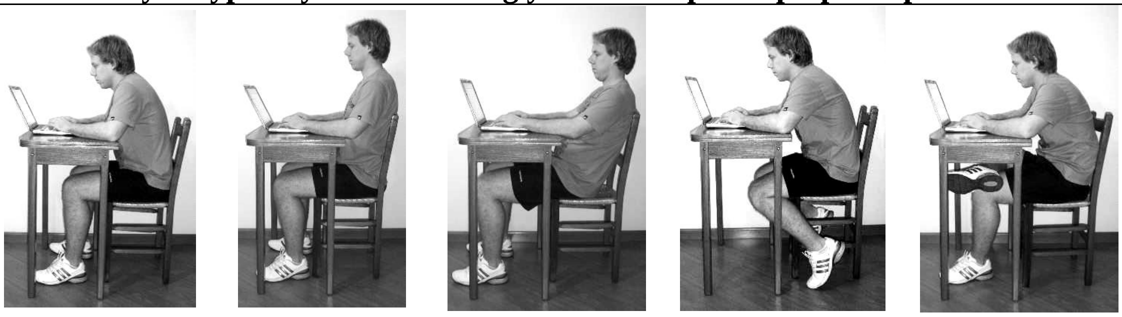

I could not identify one among these

☐ ☐ ☐ ☐ ☐ ☐

13. How do you typically sit when using your cellphone/tablet?

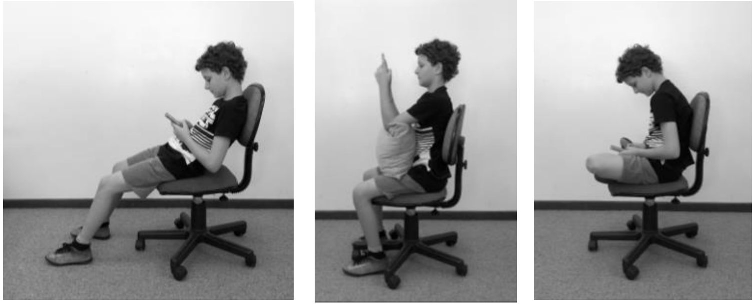

I could not identify one among these

☐ ☐ ☐ ☐

14. How do you typically use your cellphone/tablet while standing?

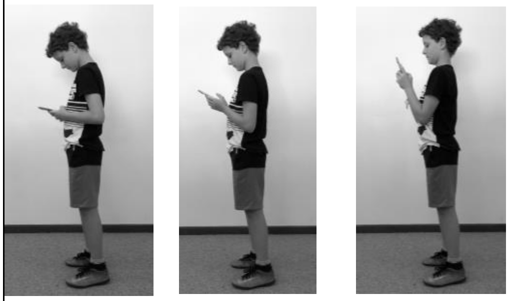

I could not identify one among these

☐ ☐ ☐ ☐

15. How do you typically pick up objects from the floor?

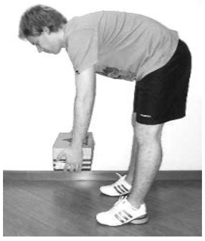
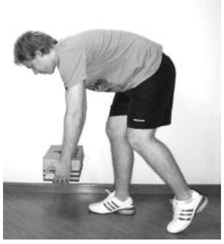
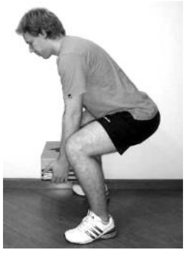
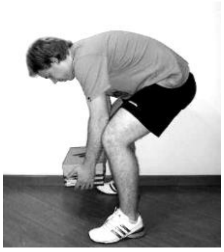

I could not identify one among these

□ □ □ □ □

16. From the picture showing below please select the image that represent the bag that you carry to the school.

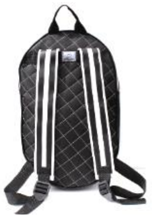	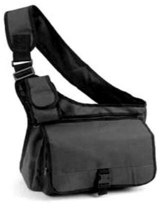	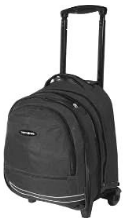	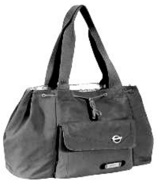	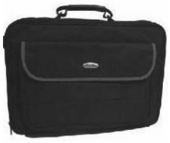	Another
Backpack with 2 straps	Backpack with 1 strap	Wheeled backpack	Bag	Briefcase	
(a)	(b)	(c)	(d)	(e)	(f)

If you chose alternative (a) (Backpack with 2 straps) **go to question 17. If not go to question 18**

17. How do you typically carry your bag to the school?

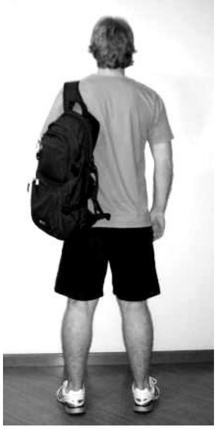
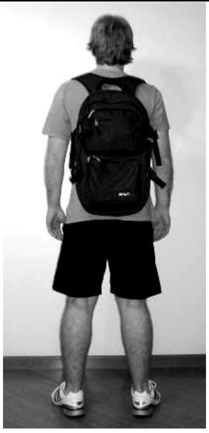
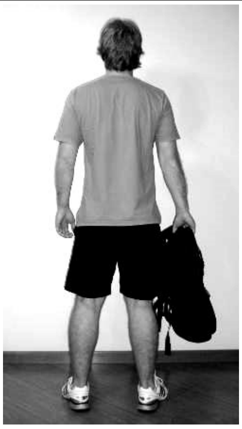
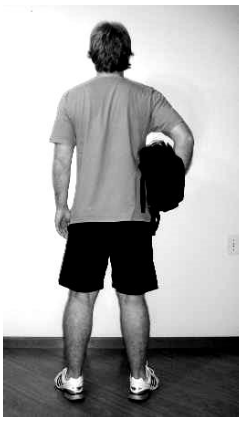
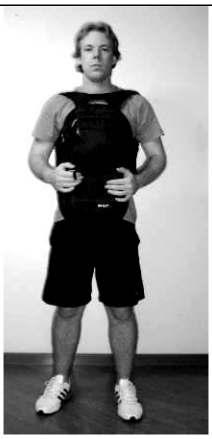

Different way/I don’t know

□ □ □ □ □ □

18. What level of formal education has your mother (or female guardian) completed?

- □ she did not attend school
- □ primary school (1st to 8th grade)
- □ secondary school (1st to 3rd grade)
- □ higher education (University)
- □ I don’t know
- □ I don’t have a female parent (guardian)

19. What level of formal education has your father (or male guardian) completed?

- □ she did not attend school
- □ primary school (1st to 8th grade)
- □ secondary school (1st to 3rd grade)
- □ higher education (University)
- □ I don’t know
- □ I don’t have a male parent (guardian)

20. Do either of your parents (or guardians) have back pain?

□ I don’t know □ yes, who?_____________ □ No

21. Have you felt (or have been) <u>back pain in the last 3 months</u>?

- ☐ yes (please continue answering the questionnaire)
- ☐ no (go to question 26)
- ☐ I don't know

22. . How often do you feel (or felt) <u>back pain</u>?

- ☐ only once
- ☐ once a month
- ☐ once a week
- ☐ more than once per week
- ☐ I don't know

23. Have you ever had to miss class because of <u>back pain</u>?

☐ yes ☐ no ☐ I don't know

24. Does the <u>back pain</u> prevent (or have prevented) you from performing daily life activities, such as: playing, practicing sports**?**

☐ yes ☐ no ☐ I don't know

25. On the scale from 0 to 10, please identify the intensity of your <u>back pain</u> for the last 3 months (Please add a "X" along the line that corresponds to your pain intensity).

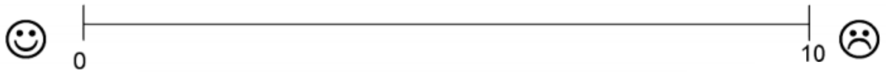

No pain The worst pain I can imagine

Questions 21 to 25 refers to the body region called "back" that is shown in this figure:

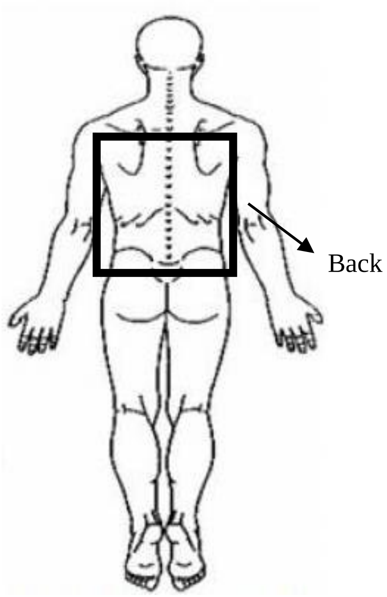

26. Have you felt (or have been) <u>neck pain</u> <u>in the last 3 months</u>?

- ☐ yes (please continue answering the questionnaire)
- ☐ no (you have finished the questionnaire, thank you)
- ☐ I don't know

27. How often do you feel (or felt) <u>neck pain</u>?

- ☐ only once
- ☐ at least once per month
- ☐ at least once per week
- ☐ more than once per week
- ☐ I don't know

28. Have you ever had to miss class because of <u>neck pain</u>?

☐ yes ☐ no ☐ I don't know

29. Does the <u>neck pain</u> prevent (or have prevented) you from performing daily life activities, such as: playing, practicing sports**?**

☐ yes ☐ no ☐ I don't know

30. On the scale from 0 to 10, please identify the intensity of your <u>neck pain</u> for the last 3 months (Please add a "X" along the line that corresponds to your pain intensity).

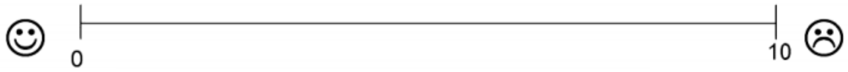

No pain The worst pain I can imagine

Questions 26 to 30 refers to the region of the body called "neck" that is shown in this figure:

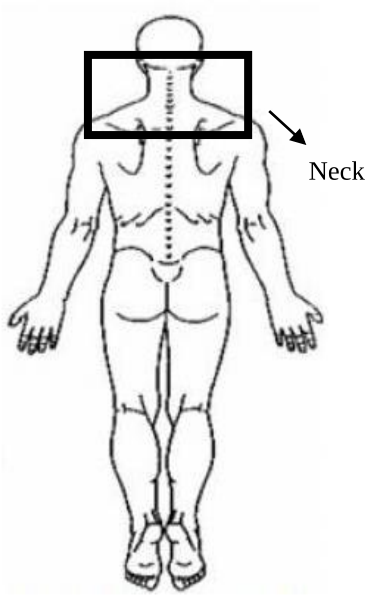

Thank you very much for your collaboration.
We really appreciate you !!!
